# Decreasing Shortest Path Length of the Sensorimotor Network Induces Frontal Glioma-Related Epilepsy

**DOI:** 10.3389/fonc.2022.840871

**Published:** 2022-02-16

**Authors:** Shengyu Fang, Lianwang Li, Shimeng Weng, Yuhao Guo, Zhong Zhang, Lei Wang, Xing Fan, Yinyan Wang, Tao Jiang

**Affiliations:** ^1^ Department of Neurosurgery, Beijing Tiantan Hospital, Capital Medical University, Beijing, China; ^2^ Beijing Neurosurgical Institute, Capital Medical University, Beijing, China; ^3^ Research Unit of Accurate Diagnosis, Treatment, and Translational Medicine of Brain Tumors, Chinese Academy of Medical Sciences, Beijing, China

**Keywords:** glioma, glioma-related epilepsy, functional network, graph theory, human

## Abstract

**Background:**

Glioma-related epilepsy (GRE) is a common symptom in patients with prefrontal glioma. Epilepsy onset is associated with functional network alterations. This study investigated alterations of functional networks in patients with prefrontal glioma and GRE.

**Methods:**

Sixty-five patients with prefrontal lobe gliomas were retrospectively assessed and classified into GRE and non-GRE groups. Additionally, 25 healthy participants were enrolled after matching for general information. Imaging data were acquired within 72 h in pre-operation. The sensorimotor network was used to delineate alterations in functional connectivity (FC) and topological properties. One-way analysis of variance and *post-hoc* analysis with Bonferroni correction were used to calculate differences of FC and topological properties.

**Results:**

All significant alterations were solely found in the sensorimotor network. Irrespective of gliomas located in the left or right prefrontal lobes, the edge between medial Brodmann area 6 and caudal ventrolateral Brodmann area 6 decreased FC in the GRE group compared with the non-GRE group [*p* < 0.0001 (left glioma), p = 0.0002 (right glioma)]. Moreover, the shortest path length decrease was found in the GRE group compared with the non-GRE group [*p* = 0.0292 (left glioma) and *p* = 0.0129 (right glioma)].

**Conclusions:**

The reduction of FC between the medial BA 6 (supplementary motor area) and caudal ventrolateral BA 6 in the ipsilateral hemisphere and the shortening of the path length of the sensorimotor network were characteristics alterations in patients with GRE onset. These findings fill in the gap which is the relationship between GRE onset and the alterations of functional networks in patients with prefrontal glioma.

**Significance Statement:**

Glioma related epilepsy is the most common symptom of prefrontal glioma. It is important to identify characteristic alterations in functional networks in patients with GRE. We found that all significant alterations occurred in the sensorimotor network. Moreover, a decreased FC in the supplementary motor area and a shortening of the path’s length are additional characteristics of glioma-related epilepsy. We believe that our findings indicate new directions of research that will contribute to future investigations of glioma-related epilepsy onset.

## Introduction

Tumor locations and low-grade glioma are susceptible factors for patients with glioma-related epilepsy (GRE) (1, 2). However, why prefrontal glioma is frequently induced GRE was still unknown. Extensive disruption of functional connectivity (FC) was thought to be related to patients with idiopathic epilepsy (3–5). Based on those studies, the investigation of epilepsy by analyzing the alterations in brain functional networks was proposed, which gives a new insight into the inspection of epilepsy.

The FC (6) and topological properties (7) are valuable to revealing the structure and conveying the ability of function networks based on resting-state functional magnetic resonance images (rs-fMRIs). Previous studies demonstrated that decreased inter-cortical FC in the different functional networks, such as sensorimotor (4), default mode (8), and visual networks (9), and decreased global efficiency were related to idiopathic epilepsy (4, 10, 11). However, different from idiopathic epilepsy only impairing functional networks, glioma not only was disrupted but also was able to reorganize functional networks (12, 13). Hence the previous conclusions that alterations of functional networks in patients with idiopathic epilepsy were not suitable for patients with glioma.

The decreasing shortest path lengths of the visual network (14) and language network (15) were markers to indicate that patients with left and right temporal lobe glioma suffered from GRE, respectively. However, it was still unknown what alterations of functional networks in patients with prefrontal glioma were related to GRE. Hence, in this study, we aimed to 1) explore how FC and topological properties alter in patients with prefrontal glioma and 2) find characteristic alterations in FC and topological properties of patients with GRE.

## Materials and Methods

The study protocol was approved by the local institutional review board.

### Participants

Between January 2016 and July 2018, 70 patients from Tiantan Hospital with a primary diagnosis of prefrontal lobe glioma were retrospectively enrolled in the study. Inclusion criteria were as follows: (a) aged older than 18 years; (b) more than 6 years of school education; (c) no history of antiepileptic drug use; and (d) no history of biopsy, radiotherapy, or chemotherapy. The exclusion criteria were as follows: (a) contraindications for MRI; (b) head motion greater than 3 mm in translation or 3° in rotation; and (c) gliomas involving bilateral prefrontal lobes or leading midline shift.

Finally, 65 patients with prefrontal gliomas were enrolled in the study (29 male and 36 female, left hemisphere n = 35, right hemisphere n= 30). All patients were divided into the GRE and non-GRE groups. Preoperative electroencephalograms (4 hours) were performed in the patients who did not have a clear history of seizures (patients in non-GRE group, n = 36). Moreover, 25 right-handed healthy participants matched to the patients for age, sex, and education level which comprised the control group (12 men and 13 female).

### Collection of Patient Clinical Characteristics

We retrospectively collected patient characteristics from inpatient records including age, sex, education level, Karnofsky performance status, histopathology, isocitrate dehydrogenase mutation status, the extent of tumor resection, information regarding preoperative seizures, type of seizure onset, and history of taking antiepileptic drugs. Follow-up information about epileptic control was obtained *via* telephone interviews at 1 year postoperatively.

### MRI Acquisition

All MR images were acquired using a MAGNETOM Prisma 3T MRI scanner (Siemens, Erlangen, Germany). T1-magnetization prepared rapid acquisition gradient echo was applied to collect anatomical images [flip angle: 8°; repetition time (TR): 2,300 ms; echo time (TE): 2.3 ms; field of view (FOV): 240 × 240 mm^2^; voxel size: 1.0 × 1.0 × 1.0 mm^3^; slice number: 192]. T2-weighted sequences were used to acquire glioma images (flip angle: 150°; TR: 5,000 ms; TE: 105 ms; FOV: 240 × 240 mm^2^; voxel size: 0.5 × 0.5 × 3 mm^3^; slice number: 33). Finally, an echo-planar imaging sequence was applied for rs-fMRI (flip angle: 75°; TR: 2,000 ms; TE: 30 ms; FOV: 220 × 220 mm^2^; voxel size: 3.0 × 3.0 × 5.0 mm^3^; slice number: 30; acquisition duration: 8 min, closed eyes during scanning). We acquired all MRI data within 72 h before tumor resection.

### Functional MRI Preprocessing

The Graph Theoretical Network Analysis software (https://www.nitrc.org/projects/gretna) (16) was applied for rs-fMRI preprocessing. For each participant, the steps of preprocessing were as follows: a) data transformation (from Digital Imaging and Communications in Medicine to Neuroimaging Informatics Technology Initiative); b) removal of the first five images; c) slice time correction; d) realignment; e) spatial normalization (normalized to echo-planar imaging template) (17); f) spatial smoothing (full width half maximum = 4 mm); g) temporal detrending (linear detrending); h) regressing out covariance (white matter signal: with WMMask_3 mm; cerebrospinal fluid signal: with CSFMask_3 mm; head motion: Friston—24 parameters); i) temporal filtering (0.01–0.1 Hz); and j) scrubbing (using default parameters and the interpolation strategy: linear interpolation, FD threshold = 0.5, previous time point number = 1, subsequent time point number = 2).

### Regions of Tumor Invasion

**Figure 1:**
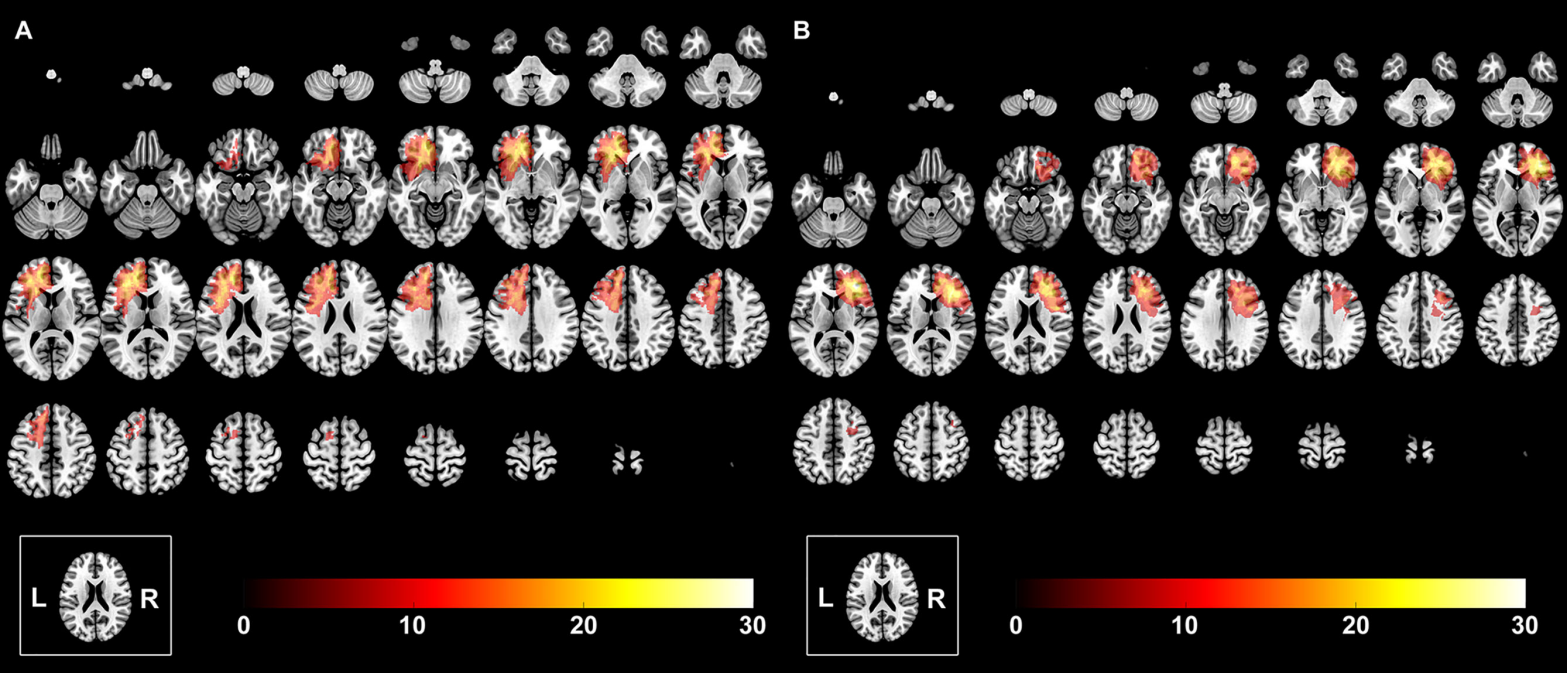
The overlapping results of prefrontal lobe gliomas. The overlapping meant that we overlapped all the tumor masks of the left hemisphere into one template to show the quantitative distribution of the left hemispheric gliomas. The value of the color bar represents the number of patients with tumor located in the same region. **(A)** Tumor was in the left hemisphere. **(B)** Tumor was in the right hemisphere.

The extent of low-grade glioma invasion was manually drawn based on the region of high signals on T2 fluid-attenuated inversion. All tumor masks were then normalized to the Montreal Neurological Institute standard space using the clinical toolbox package in SPM8 (http://www.fil.ion.ucl.ac.uk/spm/software/spm8/), which are shown in **Figure 1**. The tumor volume was calculated with the volumetric method based on the individual tumor mask.

### Regions of Interest

To calculate FC within cerebral functional networks, regions of interest (ROIs) were extracted from an open-access brain atlas, “brainnetome atlas” (http://www.brainnetome.org/) (18), which comprises 246 brain regions. To avoid the possibility that tumor invasion may affect the results of FC, the subnetworks that were invaded by gliomas and glioma-related edema were not analyzed. Therefore, in the current study, sub-templates were extracted, including the sensorimotor, visual, auditory, and left/right executive networks (based on tumor location). Details of ROIs are provided in **Tables S1**–**S5**.

### Network Construction

Pearson’s correlation coefficients were used to compare regional mean time series for all possible pairs of nodes that constructed the FC matrix. Consequently, four different FC matrices were generated from the four sub-templates of the sensorimotor, visual, auditory, and left/right executive networks.

### Graph Theoretical Measures

Global and nodal topological properties, including cluster coefficient, global efficiency, the shortest path length, local efficiency, nodal efficiency, nodal local efficiency, and small-worldness properties, were calculated for the GRE, non-GRE, and healthy groups using the graph theory analysis (19). All matrices were transformed into absolute value matrices before calculating topological properties. A total of 10,000 random networks were used to calculate the topological properties.

Small-worldness properties, including gamma, lambda, and sigma, represent the efficiency of information delivery: gamma (γ) = C_real_/C_random_ >> 1 (C represented cluster coefficient), lambda (λ) = L_real_/L_random_ ~ 1 (L represented shortest path length), and sigma (σ) = γ/λ > 1 (20). A high value of sigma represents a high efficiency of information delivery.

### Statistical Analyses

We compared the GRE, non-GRE, and healthy groups to identify which alterations were caused by each factor using SPSS (19.0 version, IBM) and GraphPad Prism 7 (GraphPad Software Inc., San Diego, USA). According to the type of data, clinical characteristics were compared between the GRE and non-GRE groups using a two-sample t-test, chi-square test, and one-way analysis of variance (ANOVA) which were used to compare among the GRE, non-GRE, and healthy groups.

When comparing the differences of FCs among the three groups, we used one-way ANOVA and *post-hoc* test with Bonferroni correction. Moreover, an eta-squared correlation was applied to explore the relationship between GRE onset and FC value.

Additionally, a series of sparsity thresholds (from 0.17 to 0.34, interval 0.01) was used to explore group differences in network topological properties, which was consistent with relevant studies (14, 15). For each property, one-way ANOVA was first applied. Subsequently, a *post-hoc* analysis with the Bonferroni correction was used if the results of ANOVA showed a significant difference (significant p-value was lower than 0.05). Clinical information (age, sex, and education level) was regressed out during statistical comparison in FC and topological properties.

## Results

### Demographic Characteristics

Based on whether patients had preoperative GRE, 29 patients were included in the GRE group (15 and 14 patients with prefrontal gliomas in the left and right hemisphere, respectively). Additionally, 36 patients comprised the non-GRE group (20 and 16 patients with prefrontal gliomas in the left and right hemispheres, respectively) (**Tables 1**, **2**). No significant differences were observed regarding age, sex, years of education, and Karnofsky performance status between the three groups. Moreover, no statistical difference in tumor volume and isocitrate dehydrogenase mutation status was found between the GRE and non-GRE groups. Furthermore, our postoperative follow-up data showed that no patient with preoperative GRE experienced epilepsy at 1 year after tumor resection. All patients achieved Engel class I.

**Table 1:** Demographic and clinical characteristics of patients with left prefrontal gliomas.

Demographic and Clinical Characteristics	GRE (n = 15)	Non-GRE (n = 20)	Health (n = 25)	*p* value
**Gender**				
Male	8	6	12	0.32
Female	7	14	13	
**Age (y)** *^a^*	39.9 ± 2.7	43.9 ± 1.7	37.9 ± 2.0	0.10
**Handness**				
Right	15	20	25	-
Left	0	0	0	-
**Preoperative KPS**				
100	14	20	25	0.14
90~100	1	0	0	
**Education level (y)** *^a^*	13.0 ± 0.8	13.2 ± 0.6	12.6 ± 0.7	0.81
**Histopathology**				
Astrocytoma	5	7	–	0.92
Oligodendroglioma	10	13	–	
**Tumor volume (mL)*^a^***	28.62 ± 3.85	26.67 ± 3.32	-	0.70
**IDH status**				
Mutation	13	16	-	0.68
Wild-type	2	4	-	
**MGMT promoter methylation**				
Methylation	11	13	-	0.72
Non-methylation	4	7	-	
**TERT promoter mutation**				
Mutation	10	11	-	0.73
Wild-type	5	9	-	
**Type of seizure**				
Secondary generalized	15	-	-	
**Period from epilepsy first onset to rs-fMRI scan (days)**	11.8 ± 1.5	-	-	-
**Frequency before diagnosis**				
Low (only once)	11	-	-	
Medium (2~3 times)	4	-	-	
**Postoperative epileptic control**				
Engel Class I	15	-	-	-

a Values are means ± standard error of mean.

KPS, Karnofsky performance status; MGMT, O^6^-methylguanine DNA methyltransferase; TERT, telomerase reverse transcriptase gene; GRE, the group of patients with glioma-related epilepsy; non-GRE, the group of patients without glioma-related epilepsy.

The two-sample t-test was used to compare tumor volume between GRE and non-GRE groups. One-way ANOVA was used to compare age, education level, and Karnofsky performance status between GRE and non-GRE groups. Chi-square tests were used to compare gender, histopathology, and IDH status between GRE and non-GRE groups.

**Table 2:** Demographic and clinical characteristics of patient with right prefrontal gliomas.

Demographic and Clinical Characteristics	GRE (n = 14)	Non-GRE (n = 16)	Health (n = 25)	*p* value
**Gender**				
Male	7	8	12	0.99
Female	7	8	13	
**Age (y)** *^a^*	37.2 ± 2.0	38.7 ± 2.2	37.9 ± 2.0	0.84
**Handness**				
Right	14	16	25	-
Left	0	0	0	-
**Preoperative KPS**				
100	12	16	25	0.07
90~100	2	0	0	
**Education level (y)** *^a^*	12.4 ± 1.0	13.6 ± 0.8	12.6 ± 0.7	0.64
**Histopathology**				
Astrocytoma	6	5	–	0.70
Oligodendroglioma	8	11		
**Tumor volume (mL)*^a^***	35.84 ± 4.53	27.62 ± 3.52	-	0.16
**IDH status**				
Mutation	12	13	-	> 0.99
Wild-type	2	3	-	
**MGMT promoter methylation**				
Methylation	9	11	-	> 0.99
Non-methylation	5	5	-	
**TERT promoter mutation**				
Mutation	7	10	-	0.71
Wild-type	7	6	-	
**Type of seizure**				
Secondary generalized	14	-	-	
**Period from epilepsy first onset to rs-fMRI scan (days)**	14.2 ± 1.2	-	-	-
**Frequency before diagnosis**				
Low (only once)	13	-	-	
Medium (2~3 times)	1	-	-	
**Postoperative epileptic control**				
Engel Class I	14	-	-	-

a Values are means ± standard error of mean.

KPS, Karnofsky performance status; MGMT, O^6^-methylguanine DNA methyltransferase; TERT, telomerase reverse transcriptase gene; GRE, the group of patients with glioma-related epilepsy; non-GRE, the group of patients without glioma-related epilepsy.

The two sample t-test was used to compare tumor volume between GRE and non-GRE groups. One-way ANOVA was used to compare age, education level, and Karnofsky performance status between GRE and non-GRE groups. Chi-square tests were used to compare gender, histopathology, and IDH status between GRE and non-GRE groups.

### Functional Connectivity Differences

The FC was compared among the GRE, non-GRE, and healthy groups in the matrices of sensorimotor, visual, auditory, and left/right executive networks (based on tumor location). Except for FC in the sensorimotor network, no significant differences in FC in the other three networks were noticed after Bonferroni correction. In total, 231 functional edges belonged to the sensorimotor network.

**Figure 2:**
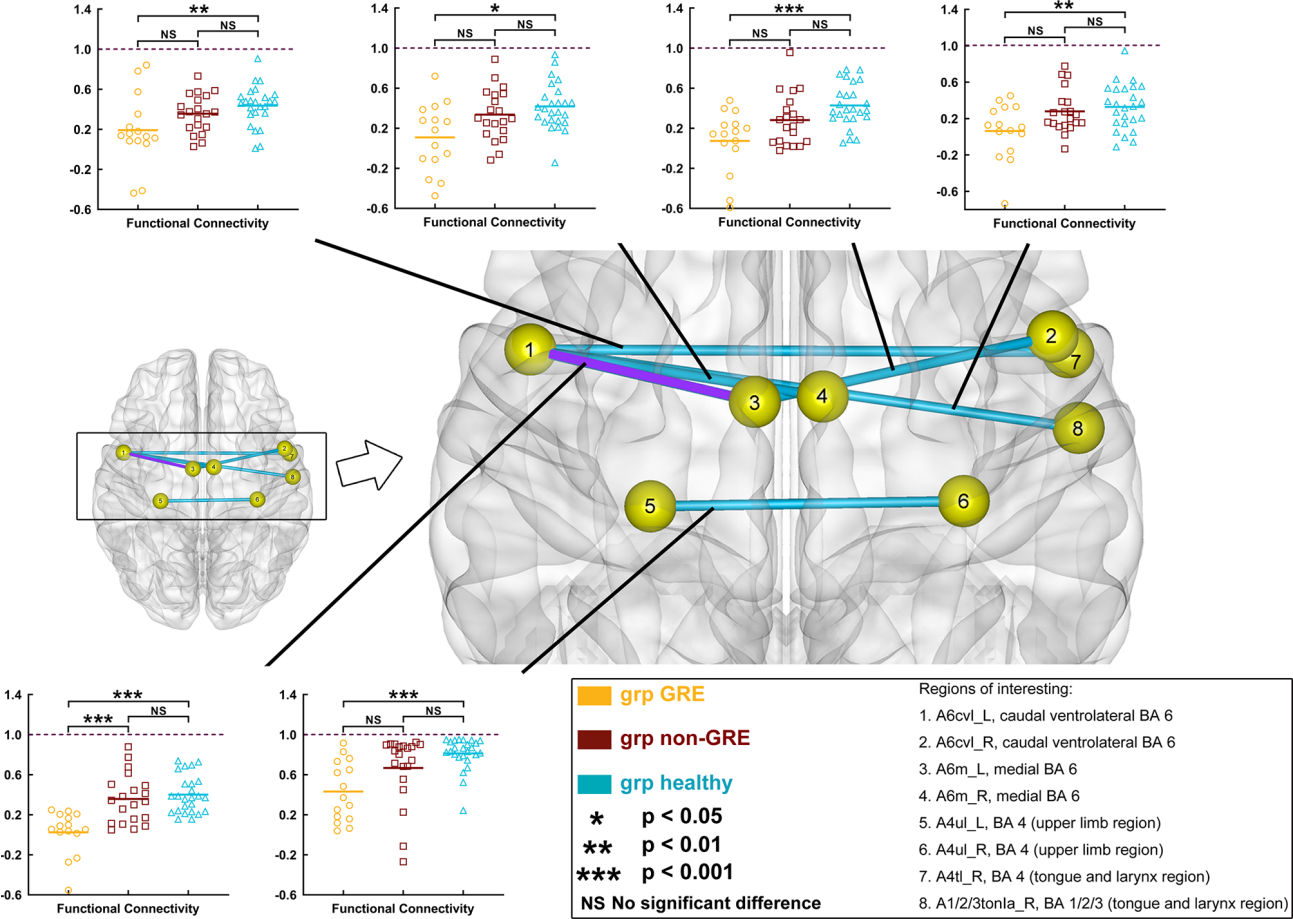
Results of alterations in functional connectivity (FC) when gliomas grew in the left prefrontal lobe. The grp GRE (n = 15), group of patients with glioma-related epilepsy. The grp non-GRE (n = 20), group of patients without glioma-related epilepsy. The grp healthy (n = 25), group of healthy participants.

When gliomas were located in the left prefrontal lobe, all these six edges were significantly different among the three groups in *post-hoc* analysis with Bonferroni correction (**Figure 2** and **Table S6**).

Compared with the non-GRE group [0.357 ± 0.055 (mean ± mean standard error)], only one edge had lower FC in the GRE group (0.025 ± 0.057) after *post-hoc* analysis with Bonferroni correction (*p* < 0.0001), which connected A6m_L [medial Brodmann area (BA) 6 in the left hemisphere] and Acvl_L (caudal ventrolateral BA 6 in the left hemisphere). Moreover, compared with the healthy group, six edges were identified with significantly lower FC in the GRE group after *post-hoc* analysis with Bonferroni correction, which connected 1) A6m_L and Acvl_L (Healthy, 0.399 ± 0.037, *p* < 0.0001); 2) Acvl_R (caudal ventrolateral BA 6 in the right hemisphere) and A6m_L (GRE, 0.108 ± 0.050; Healthy, 0.418 ± 0.047, *p* = 0.0027); 3) A6m_R (medial BA 6 in the right hemisphere) and Acvl_L (GRE, 0.074 ± 0.064; Healthy, 0.428 ± 0.043, *p* = 0.0003); 4) A4tl_R (tongue and larynx region in the right hemisphere) and Acvl_L (GRE, 0.131 ± 0.047; Healthy, 0.436 ± 0.040, *p* = 0.0069); 5) A1/2/3tonla_R (tongue and larynx region of BA 1/2/3 in the right hemisphere) and Acvl_L (GRE, 0.063 ± 0.061; Healthy, 0.329 ± 0.050, *p* = 0.0091); and 6) A4ul_L (upper limb region of BA 4 in the left hemisphere) and A4ul_R (upper limb region of BA 4 in the right hemisphere) (GRE, 0.431 ± 0.077; Healthy, 0.812 ± 0.057, *p* = 0.0009). Moreover, no edge of FC was significantly different between the non-GRE and healthy groups.

**Figure 3:**
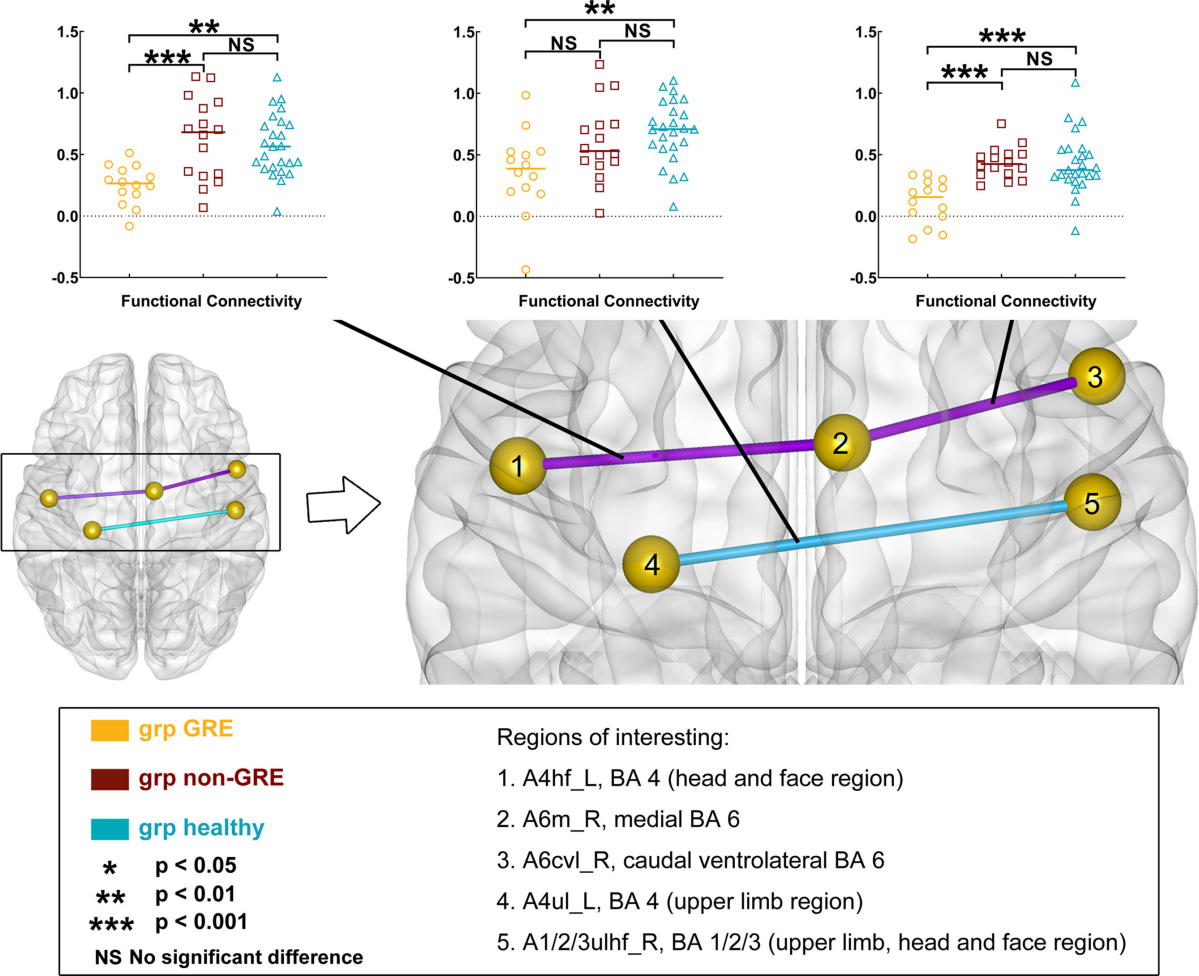
Results of alterations in functional connectivity (FC) when gliomas grew in the right prefrontal lobe. The grp GRE (n = 14), group of patients with glioma-related epilepsy. The grp non-GRE (n = 16), group of patients without glioma-related epilepsy. The grp healthy (n = 25), group of healthy participants.

When gliomas were located in the right prefrontal lobe, three edges of FC were significant different among the three groups in *post-hoc* analysis with Bonferroni test (**Figure 3** and **Table S7**).

Compared with the non-GRE group, two edges that were from A6m_R to Acvl_R (GRE, 0.118 ± 0.048; non-GRE, 0.437 ± 0.033, *p* = 0.0002) and A6m_R to A4hf_L (head and face regions of BA 4 in the left hemisphere; GRE, 0.252 ± 0.042; non-GRE, 0.657 ± 0.083, *p* = 0.0007) were identified with significantly lower FC in the GRE group. Moreover, compared with the healthy group, three edges were identified with significantly lower FC in the GRE group, which connected 1) A6m_R and Acvl_R (Healthy, 0.429 ± 0.048, *p* < 0.0001); 2) A4hf_L and A6m_R (Healthy, 0.575 ± 0.050, *p* = 0.0014); and 3) A1/2/3ulhf_R (upper limb, head, and face regions of BA 1/2/3 in the right hemisphere) and A4ul_L (GRE, 0.359 ± 0.089; Healthy, 0.691 ± 0.051, *p* = 0.0042). Additionally, no edge of FC was significantly different between the non-GRE and healthy groups.

### The Relationship Between Functional Connectivity and Occurring GRE

Our results showed a negative correlation between FC of the functional edge connected A6m_L to Acvl_L when the glioma was located in the left hemisphere (r = -0.590, *p* < 0.0001, eta-squared correlation). A similar result was found when the glioma was located in the right hemisphere (functional edge: connected A6m_R and Acvl_R, r = -0.541, *p* < 0.0001).

### Differences in Global Topological Properties

**Figure 4:**
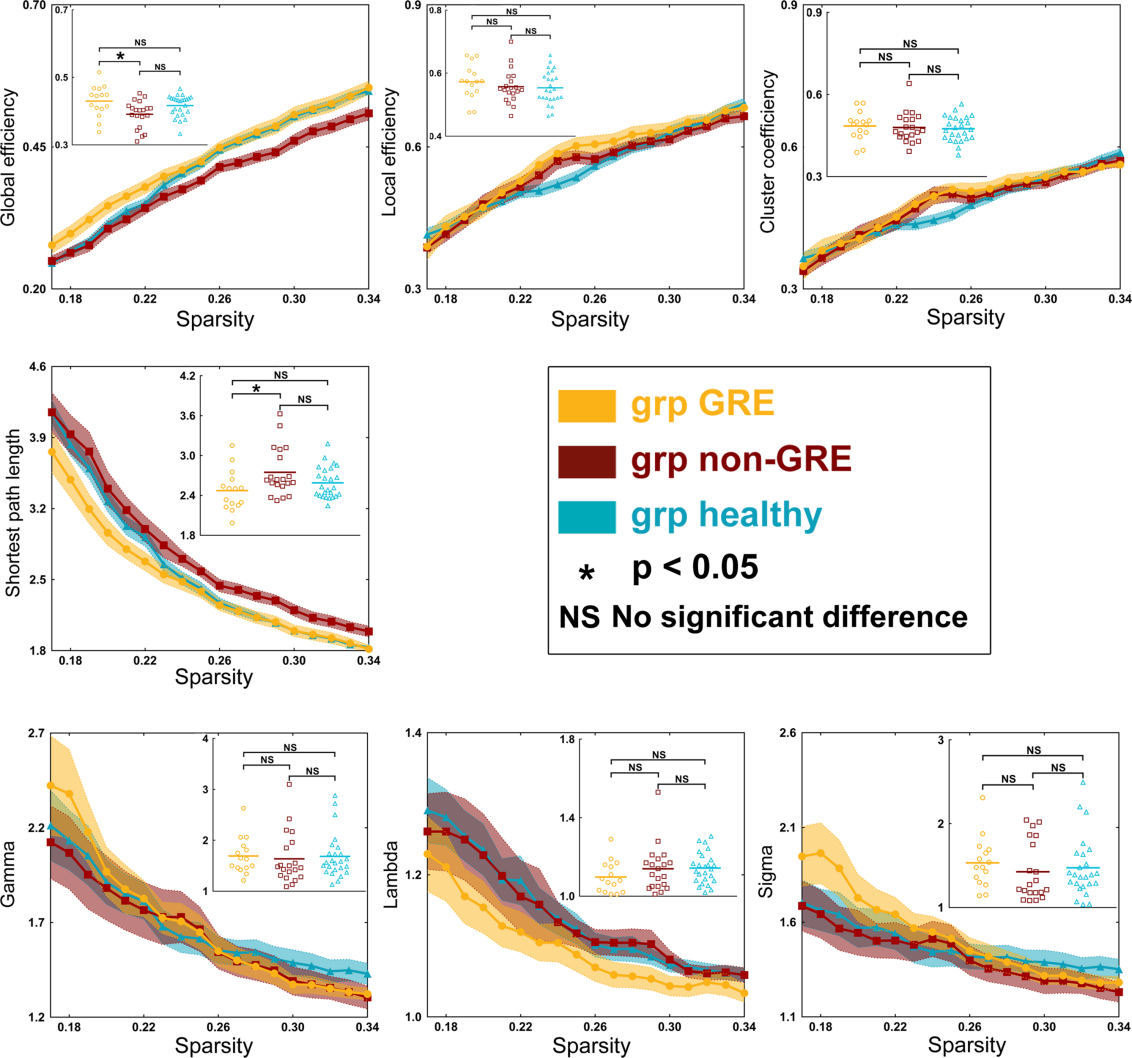
Results of alterations in topological properties when gliomas grew in the left prefrontal lobe. Each property was analyzed with one-way ANOVA test and *post-hoc* test with least significant difference correction. The grp GRE (n = 15), group of patients with glioma-related epilepsy. The grp non-GRE (n = 20), group of patients without glioma-related epilepsy. The grp healthy (n = 25), group of healthy participants.

When gliomas were located in the left prefrontal hemisphere, there were some differences in global efficiency (*p* = 0.0118) and shortest path length (*p* = 0.0306) among the three groups in the sensorimotor network in one-way ANOVA (**Table S8** and **Figure 4**). After *post-hoc* analysis with Bonferroni correction, the non-GRE group (0.391 ± 0.009) showed weaker global efficiency than the GRE group (0.430 ± 0.012, *p* = 0.0113). Moreover, compared with the non-GRE group (2.747 ± 0.081), the shortest path length was significantly shorter in the GRE group (2.473 ± 0.079, *p* = 0.0292).

**Figure 5:**
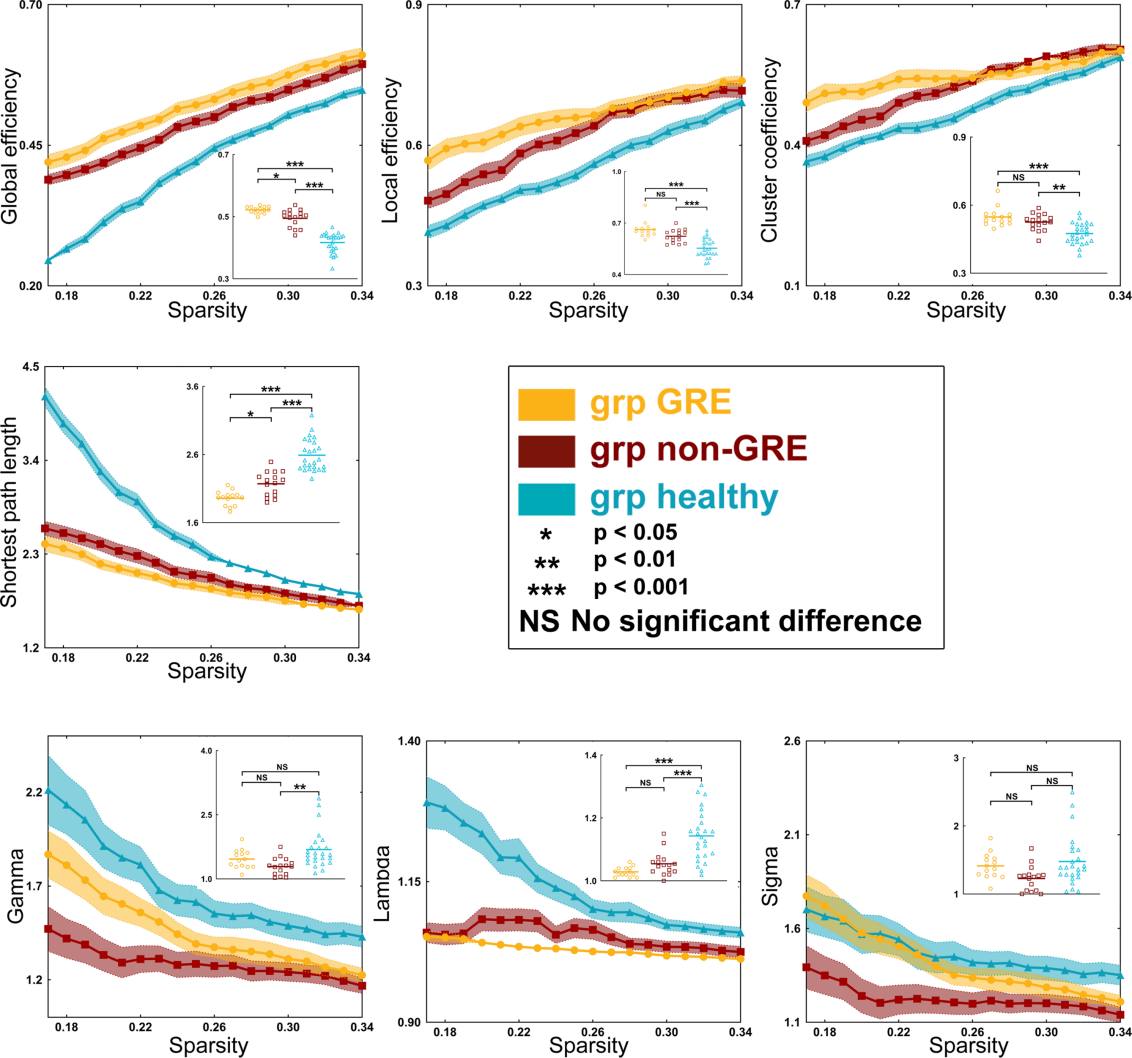
Results of alterations in topological properties gliomas grew in the right prefrontal lobe. Each property was analyzed with one-way ANOVA test and *post-hoc* test with least significant difference correction. The grp GRE (n = 14), group of patients with glioma-related epilepsy. The grp non-GRE (n = 16), group of patients without glioma-related epilepsy. The grp healthy (n = 25), group of healthy participants.

When gliomas were located in the right prefrontal lobe, there were some differences in global efficiency (*p* < 0.0001), shortest path length (*p* < 0.0001), local efficiency (*p* < 0.0001), and clustering coefficient (*p* < 0.0001) among the three groups in the sensorimotor network by one-way ANOVA (**Table S9** and **Figure 5**). After a *post-hoc* analysis with Bonferroni correction, in the GRE group (0.521 ± 0.003), the global efficiency was significantly greater than those in the non-GRE group (non-GRE, 0.494 ± 0.007, *p* = 0.0214). Additionally, the shortest path length in the GRE group (1.958 ± 0.029) was shorter than in the non-GRE group (non-GRE, 2.170 ± 0.044, *p* = 0.0129). Moreover, compared with the healthy group, the GRE and non-GRE groups showed significantly greater global efficiency (hHealthy = 0.416 ± 0.006; GRE vs. healthy, *p* < 0.0001; and non-GRE vs. healthy, *p* < 0.0001), local efficiency (GRE = 0.662 ± 0.013, non-GRE = 0.624 ± 0.010, Healthy = 0.554 ± 0.010, GRE vs. healthy, *p* < 0.0001, and non-GRE vs. healthy, *p* < 0.0001), and clustering coefficient (GRE = 0.548 ± 0.012, non-GRE = 0.525 ± 0.009, Healthy = 0.475 ± 0.009; GRE vs. healthy, *p* < 0.0001, and non-GRE vs. healthy, *p* = 0.0012) after *post-hoc* analysis with Bonferroni correction. The shortest path length was significantly shorter in the GRE and non-GRE groups than in the healthy group (GRE, 1.959 ± 0.029, non-GRE, 2.170 ± 0.044, Healthy, 2.589 ± 0.047; GRE vs. healthy groups, *p* < 0.0001, and non-GRE vs. healthy groups, *p* < 0.0001).

In addition, the gamma (*p* = 0.0021) and lambda (*p* < 0.0001) values showed a significant difference among the three groups in one-way ANOVA. Furthermore, after the *post-hoc* analysis with the Bonferroni correction test, the value of gamma was found significantly higher in the healthy group (1.684 ± 0.088) than that in the non-GRE group (1.288 ± 0.050, *p* = 0.0017). The value of lambda was found significantly higher in the healthy group (1.143 ± 0.016) than in the GRE (1.029 ± 0.004, *p* < 0.0001) and non-GRE groups (1.054 ± 0.009, p < 0.0001).

No difference of global topological properties in the other three networks (visual, auditory, and left/right executive) was found among the three groups.

### Differences in Nodal Topological Properties

We analyzed the nodal efficiency and nodal local efficiency among the three groups (**Tables S10**, **S11**) whether the gliomas were located in the left or right hemisphere. However, no significant difference was found among these three groups in nodal properties by one-way ANOVA. Moreover, no difference in nodal topological properties was found among these three groups in the other three networks (visual, auditory, and left/right executive).

## Discussion

This study investigated the characteristic alterations of functional networks in patients with prefrontal glioma and GRE. Our findings implied that gliomas located in different hemispheres may alter FC and topological properties in different ways. In addition, decreasing FC between ipsilateral medial BA 6 (supplementary motor area) and caudal ventrolateral BA 6 and shortening path length of the whole sensorimotor network were characteristic alterations in patients with GRE.

Seizure onset is associated with alterations in functional networks (14). In our previous studies, we found that the decreasing shortest path length was a marker to indicate that patients with temporal lobe glioma suffered from GRE. Nevertheless, the relationship between GRE onset and alterations of functional networks in patients with prefrontal glioma remains unclear. Whether glioma in the left or right prefrontal lobe, the edge connecting the medial BA 6 and caudal ventrolateral BA 6 areas in the ipsilateral hemisphere reduced their FC in the GRE group but insignificantly altered in the non-GRE group. The medial BA 6 area belongs to the supplementary motor area and the caudal ventrolateral BA 6 area belongs to the premotor area which is responsible for integrating and regulating motor information that controls the primary motor area to generate movement (21, 22). Moreover, our results of correlation analysis indicated a significant negative correlation between GRE onset and FC of this edge. Hence, we thought that glioma led some patients to decrease their FC in the BA 6 area, and this alteration was specifically related to GRE onset. Due to the FC reduction in the BA 6 area, the ability of motor functional control in the supplementary motor area is impaired. Hence, this alteration is presented in patients who suffer from GRE more easily. Simultaneously, this finding explained why the GRE was associated with their gliomas growing in or adjacent to the premotor area (23).

In our study, we found that the number of edges with FC significant alteration was different between the left and right gliomas. We thought that this difference was related to tumor locations. Our previous findings indicated that the left temporal glioma alters the visual network (14) but the right temporal glioma alters the language network (15). It meant different tumor location alters different networks. Hence, in this study, only the sensorimotor network was altered by prefrontal glioma, but the alterations of edges were different. For this reason, we divided all patients into four groups based on the history of GRE and tumor location instead of making the tumor into the same hemisphere through flipping the rs-fMRI data like in a previous study (24).

An increase in the shortest path length was widely observed in patients with idiopathic epilepsy (25, 26). This change was related to long-term and frequent epilepsy onset that caused cortical sclerosis (27), gray matter atrophy (28), and cortical hypo-metabolism (29). These alterations of gray matter induce the pathways of information conveying disruption. However, different from idiopathic epilepsy, preoperative GRE was a relatively short-term symptom, since the glioma is being removed as soon as possible once the GRE appears, and most of GRE onset will be controlled (1). Hence, our findings that the shortest path length decreased and global efficiency increased in the GRE group were conversely to idiopathic epilepsy.

The alterations of topological property in glioma patients were related to network reorganization. Previous studies showed that glioma can induce network reorganization in cortices that are close to the lesion (30, 31). In the current study, all gliomas were located in the prefrontal lobe that was near the sensorimotor network (especially near the supplementary motor area and premotor area). Hence, the topological properties of the sensorimotor network altered. For this reason, the FC of the edge between supplementary motor area and premotor area also altered.

Glioma-induced reorganization resulted in the shortest path length of sensorimotor networks being shortened (32, 33). A shorter network path contributed to a reduction in the convulsive threshold underlying epileptic seizures (34, 35). Conversely, longer network paths contributed to prolonging systematic response time that counteracted the rapid spread of local epileptic discharges (36). Compared with the non-GRE group, the patients in the GRE group have more significantly altered in the shortest path length. Thus, we thought that the shortened path length of the sensorimotor network was related to GRE onset.

Interestingly, the alterations of topological properties in patients with different hemispheric glioma were a little different when comparing the healthy group with the non-GRE group. We inferred that this discrepancy might be related to two reasons. On the one hand, all patients and healthy subjects in this study were right handed. Hence, different topological properties of sensorimotor network might exist between the left and right hemisphere. The left hemispheric motor cortices control right-sided motor functions, which were used more frequently and were more flexible than left-sided motor functions (37, 38). On the other hand, there might be some bias in selection since the sample size was relevant small, especially for patients with right gliomas.

Even though the number of patients was limited, the positive results identified in our study were reliable under strict statistical corrections. Here, the topological properties were calculated using functional matrices with absolute values as in previous studies (20, 39, 40). Moreover, all patients in our study did not use antiepileptic drugs before preoperative rs-fMRI scanning. Hence, we could not investigate how antiepileptic drugs could influence functional network alterations. Furthermore, the number of patients with other type of seizure was too small to analysis. Hence, we only investigated the patients with secondary generalized GRE in this study. In the future, we will enroll more patients to validate our findings.

In summary, the reduction of FC between the medial BA 6 (supplementary motor area) and caudal ventrolateral BA 6 in the ipsilateral hemisphere and a shortening of the path length of the sensorimotor network were characteristics alterations in patients with GRE onset. These findings reveal the relationship between GRE onset and alterations in brain functional networks in patients with prefrontal glioma.

## Data Availability Statement

The original contributions presented in the study are included in the article/**Supplementary Material**. Further inquiries can be directed to the corresponding authors.

## Ethics Statement

The studies involving human participants were reviewed and approved by the IRB of Beijing Tiantan Hospital. The patients/participants provided their written informed consent to participate in this study.

## Author Contributions

Study concept and design: SF, LL, SW, and YG. Data acquisition and analysis: SF, LL, SW, ZZ, and YG. Statistics/verification of the analytical method: SF, XF, and YW. Writing of the first draft: SF, XF, LW, and YW. Supervision study: XF, YW, and TJ. All authors contributed to the article and approved the submitted version.

## Funding

This work was supported by the Public Welfare Development and Reform Pilot Project of Beijing Medical Research Institute (PXM2019_026280_000008), Beijing Municipal Natural Science Foundation (No. 7202021), National Natural Science Foundation of China (No. 82001777), and Research Unit of Accurate Diagnosis, Treatment, and Translational Medicine of Brain Tumors Chinese (No. 2019-I2M-5-021).

## Conflict of Interest

The authors declare that the research was conducted in the absence of any commercial or financial relationships that could be construed as a potential conflict of interest.

## Publisher’s Note

All claims expressed in this article are solely those of the authors and do not necessarily represent those of their affiliated organizations, or those of the publisher, the editors and the reviewers. Any product that may be evaluated in this article, or claim that may be made by its manufacturer, is not guaranteed or endorsed by the publisher.

## References

[1] You G Sha Z Jiang T. Clinical Diagnosis and Perioperative Management of Glioma-Related Epilepsy. Front Oncol (2020) 10:550353. doi: 10.3389/fonc.2020.550353 33520690 PMC7841407

[2] Liang S Fan X Zhao M Shan X Li W Ding P. Clinical Practice Guidelines for the Diagnosis and Treatment of Adult Diffuse Glioma-Related Epilepsy. Cancer Med (2019) 8(10):4527–35. doi: 10.1002/cam4.2362 PMC6712518 31240876

[3] Luo C Li Q Xia Y Lei X Xue K Yao Z. Resting State Basal Ganglia Network in Idiopathic Generalized Epilepsy. Hum Brain Mapp (2012) 33(6):1279–94. doi: 10.1002/hbm.21286 PMC6869872 21520351

[4] Li Q Chen Y Wei Y Chen S Ma L He Z. Functional Network Connectivity Patterns Between Idiopathic Generalized Epilepsy With Myoclonic and Absence Seizures. Front Comput Neurosci (2017) 11:38. doi: 10.3389/fncom.2017.00038 28588471 PMC5440462

[5] Zhong C Liu R Luo C Jiang S Dong L Peng R. Altered Structural and Functional Connectivity of Juvenile Myoclonic Epilepsy: An fMRI Study. Neural Plast (2018) 2018:7392187. doi: 10.1155/2018/7392187 29681927 PMC5846383

[6] Negishi M Martuzzi R Novotny EJ Spencer DD Constable RT. Functional MRI Connectivity as a Predictor of the Surgical Outcome of Epilepsy. Epilepsia (2011) 52(9):1733–40. doi: 10.1111/j.1528-1167.2011.03191.x PMC3169719 21801165

[7] Bernhardt BC Chen Z He Y Evans AC Bernasconi N. Graph-Theoretical Analysis Reveals Disrupted Small-World Organization of Cortical Thickness Correlation Networks in Temporal Lobe Epilepsy. Cereb Cortex (2011) 21(9):2147–57. doi: 10.1093/cercor/bhq291 21330467

[8] Yang T Luo C Li Q Guo Z Liu L Gong Q. Altered Resting-State Connectivity During Interictal Generalized Spike-Wave Discharges in Drug-Naive Childhood Absence Epilepsy. Hum Brain Mapp (2013) 34(8):1761–7. doi: 10.1002/hbm.22025 PMC6870260 22431250

[9] Cao X Qian Z Xu Q Shen J Zhang Z Lu G. Altered Intrinsic Connectivity Networks in Frontal Lobe Epilepsy: A Resting-State fMRI Study. Comput Math Methods Med (2014) 2014:864979. doi: 10.1155/2014/864979 25525456 PMC4261631

[10] Liao W Zhang Z Pan Z Mantini D Ding J Duan X. Altered Functional Connectivity and Small-World in Mesial Temporal Lobe Epilepsy. PloS One (2010) 5(1):e8525. doi: 10.1371/journal.pone.0008525 20072616 PMC2799523

[11] Roland JL Snyder AZ Hacker CD Mitra A Shimony JS Limbrick DD. On the Role of the Corpus Callosum in Interhemispheric Functional Connectivity in Humans. Proc Natl Acad Sci USA (2017) 114(50):13278–83. doi: 10.1073/pnas.1707050114 PMC5740665 29183973

[12] Duffau H. Mapping the Connectome in Awake Surgery for Gliomas: An Update. J Neurosurg Sci (2017) 61(6):612–30. doi: 10.23736/S0390-5616.17.04017-6 28263047

[13] Tate MC Herbet G Moritz-Gasser S Tate JE Duffau H. Probabilistic Map of Critical Functional Regions of the Human Cerebral Cortex: Broca's Area Revisited. Brain (2014) 137(Pt 10):2773–82. doi: 10.1093/brain/awu168 24970097

[14] Fang S Zhou C Fan X Jiang T Wang Y. Epilepsy-Related Brain Network Alterations in Patients With Temporal Lobe Glioma in the Left Hemisphere. Front Neurol (2020) 11:684. doi: 10.3389/fneur.2020.00684 32765403 PMC7380082

[15] Fang S Wang Y Jiang T. Epilepsy Enhance Global Efficiency of Language Networks in Right Temporal Lobe Gliomas. CNS Neurosci Ther (2021) 27(3):363–71. doi: 10.1111/cns.13595 PMC7871790 33464718

[16] Wang J Wang X Xia M Liao X Evans A He Y. GRETNA: A Graph Theoretical Network Analysis Toolbox for Imaging Connectomics. Front Hum Neurosci (2015) 9:386. doi: 10.3389/fnhum.2015.00386 26175682 PMC4485071

[17] Calhoun VD Wager TD Krishnan A Rosch KS Seymour KE Nebel MB. The Impact of T1 Versus EPI Spatial Normalization Templates for fMRI Data Analyses. Hum Brain Mapp (2017) 38(11):5331–42. doi: 10.1002/hbm.23737 PMC5565844 28745021

[18] Fan L Li H Zhuo J Zhang Y Wang J Chen L. The Human Brainnetome Atlas: A New Brain Atlas Based on Connectional Architecture. Cereb Cortex (2016) 26(8):3508–26. doi: 10.1093/cercor/bhw157 PMC4961028 27230218

[19] Hart MG Ypma RJ Romero-Garcia R Price SJ Suckling J. Graph Theory Analysis of Complex Brain Networks: New Concepts in Brain Mapping Applied to Neurosurgery. J Neurosurg (2016) 124(6):1665–78. doi: 10.3171/2015.4.JNS142683 26544769

[20] Gong Y Wu H Li J Wang N Liu H Tang X. Multi-Granularity Whole-Brain Segmentation Based Functional Network Analysis Using Resting-State fMRI. Front Neurosci (2018) 12:942. doi: 10.3389/fnins.2018.00942 30618571 PMC6299028

[21] Vassal M Charroud C Deverdun J Le Bars E Molino F Bonnetblanc F. Recovery of Functional Connectivity of the Sensorimotor Network After Surgery for Diffuse Low-Grade Gliomas Involving the Supplementary Motor Area. J Neurosurg (2017) 126:1181–90. doi: 10.3171/2016.4.JNS152484 27315027

[22] Fang S Li Y Wang Y Zhang Z Jiang T. Awake Craniotomy for Gliomas Involving Motor-Related Areas: Classification and Function Recovery. J Neurooncol (2020) 148(2):317–25. doi: 10.1007/s11060-020-03520-w 32350781

[23] Wang Y Qian T You G Peng X Chen C You Y. Localizing Seizure-Susceptible Brain Regions Associated With Low-Grade Gliomas Using Voxel-Based Lesion-Symptom Mapping. Neuro Oncol (2015) 17(2):282–8. doi: 10.1093/neuonc/nou130 PMC4288515 25031032

[24] Vassal M Charroud C Deverdun J Le Bars E Molino F Bonnetblanc F. Recovery of Functional Connectivity of the Sensorimotor Network After Surgery for Diffuse Low-Grade Gliomas Involving the Supplementary Motor Area. J Neurosurg (2017) 126(4):1181–90. doi: 10.3171/2016.4.JNS152484 27315027

[25] Besseling RM Overvliet GM Jansen JF van der Kruijs SJ Vles JS Ebus SC. Aberrant Functional Connectivity Between Motor and Language Networks in Rolandic Epilepsy. Epilepsy Res (2013) 107(3):253–62. doi: 10.1016/j.eplepsyres.2013.10.008 24210960

[26] Lee C Im CH Koo YS Lim JA Kim TJ Byun JI. Altered Network Characteristics of Spike-Wave Discharges in Juvenile Myoclonic Epilepsy. Clin EEG Neurosci (2017) 48(2):111–7. doi: 10.1177/1550059415621831 26697882

[27] Aparicio J Carreno M Bargallo N Setoain X Rubi S Rumia J. Combined (18)F-FDG-PET and Diffusion Tensor Imaging in Mesial Temporal Lobe Epilepsy With Hippocampal Sclerosis. NeuroImage Clin (2016) 12:976–89. doi: 10.1016/j.nicl.2016.05.002 PMC5153605 27995064

[28] Caciagli L Bernasconi A Wiebe S Koepp MJ Bernasconi N Bernhardt BC. A Meta-Analysis on Progressive Atrophy in Intractable Temporal Lobe Epilepsy: Time Is Brain? Neurology (2017) 89(5):506–16. doi: 10.1212/WNL.0000000000004176 PMC5539734 28687722

[29] Celiker Uslu S Yuksel B Tekin B Sariahmetoglu H Atakli D. Cognitive Impairment and Drug Responsiveness in Mesial Temporal Lobe Epilepsy. Epilepsy Behav (2019) 90:162–7. doi: 10.1016/j.yebeh.2018.10.034 30576963

[30] Pillai JJ. Insights Into Adult Postlesional Language Cortical Plasticity Provided by Cerebral Blood Oxygen Level-Dependent Functional MR Imaging. AJNR Am J Neuroradiol (2010) 31(6):990–6. doi: 10.3174/ajnr.A1896 PMC7963935 20007726

[31] Guerra-Carrillo B Mackey AP Bunge SA. Resting-State fMRI: A Window Into Human Brain Plasticity. Neuroscientist (2014) 20(5):522–33. doi: 10.1177/1073858414524442 24561514

[32] van Dokkum LEH Moritz Gasser S Deverdun J Herbet G Mura T D'Agata B. Resting State Network Plasticity Related to Picture Naming in Low-Grade Glioma Patients Before and After Resection. NeuroImage Clin (2019) 24:102010. doi: 10.1016/j.nicl.2019.102010 31734532 PMC6861733

[33] Laura G Silvia T Nikolaos P Patrizia P. The Role of fMRI in the Assessment of Neuroplasticity in MS: A Systematic Review. Neural Plast (2018) 2018:3419871. doi: 10.1155/2018/3419871 30693023 PMC6332922

[34] Ponten SC Bartolomei F Stam CJ. Small-World Networks and Epilepsy: Graph Theoretical Analysis of Intracerebrally Recorded Mesial Temporal Lobe Seizures. Clin Neurophysiol (2007) 118(4):918–27. doi: 10.1016/j.clinph.2006.12.002 17314065

[35] Dyhrfjeld-Johnsen J Santhakumar V Morgan RJ Huerta R Tsimring L Soltesz I. Topological Determinants of Epileptogenesis in Large-Scale Structural and Functional Models of the Dentate Gyrus Derived From Experimental Data. J Neurophysiol (2007) 97(2):1566–87. doi: 10.1152/jn.00950.2006 17093119

[36] Ofer I LeRose C Mast H LeVan P Metternich B Egger K. Association Between Seizure Freedom and Default Mode Network Reorganization in Patients With Unilateral Temporal Lobe Epilepsy. Epilepsy Behav (2019) 90:238–46. doi: 10.1016/j.yebeh.2018.10.025 30538081

[37] Rosazza C Deleo F D'Incerti L Antelmi L Tringali G Didato G. Tracking the Re-Organization of Motor Functions After Disconnective Surgery: A Longitudinal fMRI and DTI Study. Front Neurol (2018) 9:400. doi: 10.3389/fneur.2018.00400 29922216 PMC5996100

[38] Sgandurra G Biagi L Fogassi L Sicola E Ferrari A Guzzetta A. Reorganization of the Action Observation Network and Sensory-Motor System in Children With Unilateral Cerebral Palsy: An fMRI Study. Neural Plast (2018) 2018:6950547. doi: 10.1155/2018/6950547 30147718 PMC6083552

[39] Fornito A Zalesky A Bullmore ET. Network Scaling Effects in Graph Analytic Studies of Human Resting-State FMRI Data. Front Syst Neurosci (2010) 4:22. doi: 10.3389/fnsys.2010.00022 20592949 PMC2893703

[40] Mazrooyisebdani M Nair VA Garcia-Ramos C Mohanty R Meyerand E Hermann B. Graph Theory Analysis of Functional Connectivity Combined With Machine Learning Approaches Demonstrates Widespread Network Differences and Predicts Clinical Variables in Temporal Lobe Epilepsy. Brain Connect (2020) 10(1):39–50. doi: 10.1089/brain.2019.0702 31984759 PMC7044761

