# Supplementary material for: Decreasing Shortest Path Length of the Sensorimotor Network Induces Frontal Glioma-Related Epilepsy
Source: Front Oncol. 2022 Feb 16;12:840871. doi: 10.3389/fonc.2022.840871 (PMC8888886; doi:10.3389/fonc.2022.840871)
Supplement: Supplementary file 1 [file DataSheet_1.pdf]

## Supplemental Tables

**Table S1. Montreal Neurological Institute (MNI) locations of 22 nodes in the sensorimotor network**

| Regions of interesting | Modified Cyto-architectonic | Left hemisphere | | | Right hemisphere | | |
|---|---|---|---|---|---|---|---|
| | | X | Y | Z | X | Y | Z |
| A6m_L(R) | Medial area BA 6 | 5 | 36 | 38 | 6 | 38 | 35 |
| A4hf_L(R) | Area BA 4 (head and face) | -49 | -8 | 39 | 55 | -2 | 33 |
| A4ul_L(R) | Area BA 4 (upper limb) | -26 | -25 | 63 | 34 | -19 | 59 |
| A4t_L(R) | Area BA 4 (trunk) | -13 | -20 | 73 | 15 | -22 | 71 |
| A4tl_L(R) | Area BA 4 (tongue and larynx) | -52 | 0 | 8 | 54 | 4 | 9 |
| A1_2_3ll_L(R) | Area BA 1/2/3 (lower limb) | -8 | -38 | 58 | 10 | -34 | 54 |
| A4ll_L(R) | Area BA 4 (lower limb) | -4 | -23 | 61 | 5 | -21 | 61 |
| A1_2_3ulhf_L(R) | Area BA 1/2/3 (upper limb and face) | -50 | -16 | 43 | 50 | -14 | 44 |
| A1_2_3tonIa_L(R) | Area BA 1/2/3 (tongue and larynx) | -56 | -14 | 16 | 56 | -10 | 15 |
| A2_L(R) | Area BA 2 | -46 | -30 | 50 | 48 | -24 | 48 |
| A1_2_3tru_L(R) | Area BA 1/2/3 (trunk) | -21 | -35 | 68 | 20 | -33 | 69 |

*****BA = Brodmann area.

**Table S2. Montreal Neurological Institute (MNI) locations of 22 nodes in the visual network.**

| Regions of interesting | Modified Cyto-architectonic | Left hemisphere | | | Right hemisphere | | |
|---|---|---|---|---|---|---|---|
| | | X | Y | Z | X | Y | Z |
| cLinG_L(R) | caudal lingual gyrus | -11 | -82 | -11 | 10 | -85 | -9 |
| rCunG_L(R) | rostral cuneus gyrus | -5 | -81 | 10 | 7 | -76 | 11 |
| cCunG_L(R) | caudal cuneus gyrus | -6 | 94 | 1 | 8 | -90 | 12 |
| rLinG_L(R) | rostral lingual gyrus | -17 | -60 | -6 | 18 | -60 | -7 |
| vmPOS_L(R) | ventromedial parietal occipital sulcus | -13 | -68 | 12 | 15 | -63 | 12 |
| mOccG_L(R) | middle occipital gyrus | -31 | -89 | 11 | 34 | -86 | 11 |
| V5/MT+_L(R) | area V5/MT+ | -46 | -74 | 3 | 48 | -70 | -1 |
| OPC_L(R) | occipital polar cortex | -18 | -99 | 2 | 22 | -97 | 4 |
| iOccG_L(R) | inferior occipital gyrus | -30 | -88 | -12 | 32 | -85 | -12 |
| msOccG_L(R) | medial superior occipital gyrus | -11 | -88 | 31 | 16 | -85 | 34 |
| lsOccG_L(R) | lateral superior occipital gyrus | -22 | -77 | 36 | 29 | -75 | 36 |

*****BA = Brodmann area.

**Table S3. Montreal Neurological Institute (MNI) locations of 6 nodes in the auditory network.**

| Regions of interesting | Modified Cyto-architectonic | Left hemisphere | | | Right hemisphere | | |
|---|---|---|---|---|---|---|---|
| | | X | Y | Z | X | Y | Z |
| A41/42_R | BA 41/42 | -54 | -32 | 12 | 54 | -24 | 11 |
| TE_R | TE1.0 and TE1.2 | -50 | -11 | 1 | 51 | -4 | -1 |
| A40rv_L(R) | rostroventral area 40 (PFop) | -53 | -31 | 23 | 55 | -26 | 26 |

*****BA = Brodmann area.

**Table S4. Montreal Neurological Institute (MNI) locations of 20 nodes in the right executive control network.**

| Regions of interesting | Modified Cyto-architectonic | Right hemisphere | | |
|---|---|---|---|---|
| | | X | Y | Z |
| A8dl_R | dorsolateral area BA 8 | 22 | 26 | 51 |
| A9m_R | medial area BA 9 | 6 | 38 | 35 |
| A9/46d_R | dorsal area BA 9/46 | 30 | 37 | 36 |
| IFJ_R | inferior frontal junction | 42 | 11 | 39 |
| A46_R | area BA 46 | 28 | 55 | 17 |
| A9/46v_R | ventral area BA 9/46 | 42 | 44 | 14 |
| A8vl_R | ventrolateral area BA 8 | 42 | 27 | 39 |
| A6vl_R | ventrolateral area BA 6 | 34 | 8 | 54 |
| A10l_R | ventrolateral area BA 10 | 25 | 61 | -4 |
| IFS_R | inferior frontal sulcus | 48 | 35 | 13 |
| A45r_R | rostral area BA 45 | 51 | 36 | -1 |
| A12/47l_R | lateral area BA 12/47 | 40 | 39 | -14 |
| A21c_R | caudal area BA 21 | 65 | -29 | -13 |
| A37vl_R | ventrolateral area BA 37 | 54 | -57 | -8 |
| A20cl_R | caudal-lateral of area BA 20 | 61 | -40 | -17 |
| A7ip_R | intraparietal area BA 7 (hIP3) | 31 | -54 | 53 |
| A39c_R | caudal area 39 (PGp) | 45 | -71 | 20 |
| A39rd_R | rostral dorsal area BA 39 (Hip3) | 39 | -65 | 44 |
| A40c_R | caudal area BA 40 (PFm) | 57 | -44 | 38 |
| A39rv_R | rostroventral area BA 39 (PGa) | 53 | -54 | 25 |

*****BA = Brodmann area. Using this template to analysis patients with left prefrontal gliomas and healthy participants.

**Table S5. Montreal Neurological Institute (MNI) locations of 20 nodes in the left executive control network.**

| Regions of interesting | Modified Cyto-architectonic | Left hemisphere | | |
|---|---|---|---|---|
| | | X | Y | Z |
| A8dl_L | dorsolateral area BA 8 | -18 | 24 | 53 |
| A9m_L | medial area BA 9 | -5 | 36 | 38 |
| A9/46d_L | dorsal area BA 9/46 | -27 | 43 | 31 |
| IFJ_L | inferior frontal junction | -42 | 13 | 36 |
| A46_L | area BA 46 | -28 | 56 | 12 |
| A9/46v_L | ventral area BA 9/46 | -41 | 41 | 16 |
| A8vl_L | ventrolateral area BA 8 | -33 | 23 | 45 |
| A6vl_L | ventrolateral area BA 6 | -32 | 4 | 55 |
| A10l_L | ventrolateral area BA 10 | -26 | 60 | -6 |
| IFS_L | inferior frontal sulcus | -47 | 32 | 14 |
| A45r_L | rostral area BA 45 | 49 | 36 | -3 |
| A12/47l_L | lateral area BA 12/47 | -41 | 32 | -9 |
| A21c_L | caudal area BA 21 | -65 | -30 | -12 |
| A37vl_L | ventrolateral area BA 37 | -55 | -60 | -6 |
| A20cl_L | caudal-lateral of area BA 20 | -59 | -42 | -16 |
| A7ip_L | intraparietal area BA 7 (hIP3) | -27 | -59 | 54 |
| A39c_L | caudal area 39 (PGp) | -34 | -80 | 29 |
| A39rd_L | rostral dorsal area BA 39 (Hip3) | -38 | -61 | 46 |
| A40c_L | caudal area BA 40 (PFm) | -56 | -49 | 38 |
| A39rv_L | rostroventral area BA 39 (PGa) | -47 | -65 | 26 |

***BA = Brodmann area. Using this template to analysis patients with right prefrontal gliomas and healthy participants.

**Table S6. Functional connections with significant group effects among the GRE, non-GRE, and health groups (gliomas in left hemisphere)**

| Connections | Functional connectivity (mean ± SEM) | | | *p* value (ANOVA test) | Bonferroni correction | | |
|---|---|---|---|---|---|---|---|
| | GRE group | non-GRE group | Health group | | GRE vs non-GRE | GRE vs healthy | non-GRE vs healthy |
| Am6_L and Acvl_L | 0.025 ± 0.057 | 0.357 ± 0.055 | 0.399 ± 0.037 | < 0.0001 | < 0.0001 | < 0.0001 | > 0.9999 |
| Acvl_L and A4tl_R | 0.131 ± 0.047 | 0.356 ± 0.042 | 0.436 ± 0.040 | 0.0097 | 0.0583 | 0.0091 | > 0.9999 |
| Acvl_L and A1/2/3tonla_R | 0.063 ± 0.061 | 0.279 ± 0.053 | 0.854 ± 0.058 | 0.0085 | 0.0850 | 0.0069 | > 0.9999 |
| Am6_R and Acvl_L | 0.074 ± 0.064 | 0.281 ± 0.057 | 0.428 ± 0.043 | 0.0005 | 0.0682 | 0.0003 | 0.9596 |
| Am6_L and Acvl_R | 0.108 ± 0.050 | 0.336 ± 0.057 | 0.418 ± 0.047 | 0.0036 | 0.0503 | 0.0027 | 0.2716 |
| A4ul_L and A4ul_R | 0.431 ± 0.077 | 0.667 ± 0.074 | 0.812 ± 0.057 | 0.0014 | 0.0783 | 0.0009 | 0.3488 |

***** BA = Brodmann area. Am6_L = medial BA 6 in the left hemisphere; Am6_R = medial BA 6 in the right hemisphere; Acvl_L = caudal ventrolateral BA 6 in the left hemisphere; Acvl_R = caudal ventrolateral BA 6 in the right hemisphere; A4tl_R = tongue and larynx region in the right hemisphere; A1/2/3tonla_R = tongue and larynx region of BA 1/2/3 in the right hemisphere; A4ul_L = upper limb region of BA 4 in the left hemisphere; A4ul_R = upper limb region of BA 4 in the right hemisphere. Threshold of Bonferroni correction was p value = $2.16 \times 10^{-4}$.

**Table S7. Functional connections with significant group effects among the GRE, non-GRE, and health groups (gliomas in right hemisphere)**

| Connections | Functional connectivity (mean ± SEM) | | | *p* value (ANOVA test) | Bonferroni correction | | |
|---|---|---|---|---|---|---|---|
| | GRE group | non-GER group | Health group | | GRE vs non-GRE | GRE vs healthy | non-GRE vs healthy |
| Am6_R and Acvl_R | 0.118 ± 0.048 | 0.437 ± 0.033 | 0.429 ± 0.048 | 0.0005 | 0.0002 | < 0.0001 | > 0.9999 |
| Am6_R and A4hf_L | 0.252 ± 0.042 | 0.626 ± 0.083 | 0.575 ± 0.050 | 0.0004 | 0.0007 | 0.0014 | > 0.9999 |
| A4ul_L and A1/2/3ulhf_R | 0.359 ± 0.089 | 0.601 ± 0.080 | 0.691 ± 0.051 | 0.0055 | 0.0861 | 0.0042 | > 0.9999 |

***** BA = Brodmann area. Am6_R = medial BA 6 in the right hemisphere; Acvl_R = caudal ventrolateral BA 6 in the right hemisphere; A4ul_L = upper limb region of BA 4 in the left hemisphere; A4hf_L = head and face region of BA 4 in the left hemisphere; A1/2/3ulhf_R = upper limb, head and face region of BA 1/2/3 in the right hemisphere. Threshold of Bonferroni correction was p value = $2.16 \times 10^{-4}$.

**Table S8. Topological properties compared between the patient and healthy groups (gliomas in the left hemisphere)**

| | GRE group | non-GRE group | Health group | One-way ANOVA (p value) | GRE vs non-GRE (p value) | GRE vs Health (p value) | non-GRE vs n Health (p value) |
|---|---|---|---|---|---|---|---|
| Global efficiency | 0.430 ± 0.012 | 0.391 ± 0.009 | 0.416 ± 0.006 | 0.0118 | 0.0133 | 0.9000 | 0.0882 |
| Local efficiency | 0.573 ± 0.014 | 0.557 ± 0.012 | 0.554 ± 0.010 | 0.5375 | - | - | - |
| Shortest path length | 2.473 ± 0.079 | 2.747 ± 0.081 | 2.589 ± 0.047 | 0.0306 | 0.0292 | 0.7191 | 0.2553 |
| Clustering coefficient | 0.485 ± 0.014 | 0.479 ± 0.012 | 0.475 ± 0.009 | 0.8242 | - | - | - |
| Gamma | 1.691 ± 0.093 | 1.636 ± 0.113 | 1.684 ± 0.088 | 0.9144 | - | - | - |
| Lambda | 1.097 ± 0.021 | 1.140 ± 0.026 | 1.143 ± 0.016 | 0.3004 | - | - | - |
| Sigma | 1.533 ± 0.078 | 1.426 ± 0.078 | 1.474 ± 0.075 | 0.6734 | - | - | - |

***** The global properties were calculated with one-way ANOVA test. If the results one-way ANOVA were significance, post-hoc analysis with least significant difference was subsequently applied.

**Table S9. Topological properties compared between the patient and healthy groups (gliomas in the right hemisphere)**

| | GRE group | non-GRE group | Health group | One-way ANOVA (p value) | GRE vs non-GRE (p value) | GRE vs Health (p value) | non-GRE vs n Health (p value) |
|---|---|---|---|---|---|---|---|
| Global efficiency | 0.522 ± 0.003 | 0.494 ± 0.007 | 0.416 ± 0.006 | < 0.0001 | 0.0214 | < 0.0001 | < 0.0001 |
| Local efficiency | 0.662 ± 0.016 | 0.624 ± 0.012 | 0.554 ± 0.010 | < 0.0001 | 0.2310 | < 0.0001 | 0.0011 |
| Shortest path length | 1.959 ± 0.029 | 2.170 ± 0.044 | 2.589 ± 0.047 | < 0.0001 | 0.0129 | < 0.0001 | < 0.0001 |
| Clustering coefficient | 0.548 ± 0.014 | 0.525 ± 0.009 | 0.475 ± 0.009 | < 0.0001 | 0.6549 | < 0.0001 | 0.0069 |
| Gamma | 1.464 ± 0.071 | 1.289 ± 0.067 | 1.684 ± 0.088 | 0.0118 | 0.7862 | 0.2927 | 0.0122 |
| Lambda | 1.029 ± 0.005 | 1.054 ± 0.014 | 1.143 ± 0.016 | < 0.0001 | > 0.9999 | < 0.0001 | 0.0010 |
| Sigma | 1.415 ± 0.064 | 1.227 ± 0.067 | 1.478 ± 0.075 | 0.5387 | - | - | - |

***** The global properties were calculated with one-way ANOVA test. If the results one-way ANOVA were significance, post-hoc analysis with least significant difference was subsequently applied.

**Table S10. Nodal efficiency compared between the patients and healthy groups**

| Node name | Glioma grew in the left prefrontal lobe | | | | Glioma grew in the right prefrontal lobe | | | |
|---|---|---|---|---|---|---|---|---|
| | GRE group | non-GRE group | Health group | One-way ANOVA (p value) | GRE group | non-GRE group | Health group | One-way ANOVA (p value) |
| A6m | 0.320 ± 0.066 | 0.376 ± 0.041 | 0.368 ± 0.034 | 0.684 | 0.522 ± 0.014 | 0.492 ± 0.013 | 0.368 ± 0.034 | 0.104 |
| A4hf | 0.498 ± 0.032 | 0.441 ± 0.027 | 0.440 ± 0.038 | 0.468 | 0.458 ± 0.054 | 0.429 ± 0.059 | 0.440 ± 0.038 | 0.924 |
| A4ul | 0.450 ± 0.026 | 0.451 ± 0.032 | 0.484 ± 0.021 | 0.569 | 0.662 ± 0.016 | 0.624 ± 0.012 | 0.484 ± 0.021 | 0.661 |
| A4t | 0.480 ± 0.029 | 0.473 ± 0.029 | 0.470 ± 0.025 | 0.973 | 0.548 ± 0.014 | 0.525 ± 0.008 | 0.470 ± 0.025 | 0.349 |
| A4tl | 0.354 ± 0.050 | 0.295 ± 0.043 | 0.317 ± 0.035 | 0.663 | 0.463 ± 0.067 | 0.288 ± 0.064 | 0.317 ± 0.035 | 0.446 |
| A1_2_3ll | 0.474 ± 0.029 | 0.364 ± 0.036 | 0.353 ± 0.030 | 0.067 | 0.229 ± 0.004 | 0.354 ± 0.012 | 0.353 ± 0.030 | 0.507 |
| A4ll | 0.482 ± 0.018 | 0.457 ± 0.019 | 0.498 ± 0.024 | 0.401 | 0.417 ± 0.061 | 0.496 ± 0.064 | 0.498 ± 0.024 | 0.804 |
| A1_2_3ulhf | 0.473 ± 0.040 | 0.491 ± 0.023 | 0.468 ± 0.025 | 0.840 | 0.595 ± 0.018 | 0.550 ± 0.052 | 0.468 ± 0.025 | 0.163 |
| A1_2_3tonIa | 0.389 ± 0.052 | 0.409 ± 0.043 | 0.423 ± 0.026 | 0.835 | 0.546 ± 0.055 | 0.576 ± 0.036 | 0.423 ± 0.026 | 0.495 |
| A2 | 0.453 ± 0.047 | 0.351 ± 0.044 | 0.412 ± 0.035 | 0.286 | 0.545 ± 0.029 | 0.577 ± 0.023 | 0.412 ± 0.035 | 0.620 |
| A1_2_3tru | 0.272 ± 0.045 | 0.294 ± 0.042 | 0.304 ± 0.035 | 0.862 | 0.456 ± 0.066 | 0.488 ± 0.068 | 0.304 ± 0.035 | 0.172 |
| A6m | 0.303 ± 0.036 | 0.239 ± 0.047 | 0.332 ± 0.030 | 0.214 | 0.504 ± 0.066 | 0.519 ± 0.032 | 0.332 ± 0.030 | 0.140 |
| A4hf | 0.171 ± 0.050 | 0.173 ± 0.040 | 0.241 ± 0.040 | 0.410 | 0.500 ± 0.051 | 0.430 ± 0.053 | 0.241 ± 0.040 | 0.176 |
| A4ul | 0.306 ± 0.047 | 0.211 ± 0.043 | 0.288 ± 0.037 | 0.265 | 0.573 ± 0.021 | 0.603 ± 0.025 | 0.288 ± 0.037 | 0.409 |
| A4t | 0.540 ± 0.025 | 0.545 ± 0.015 | 0.522 ± 0.022 | 0.730 | 0.477 ± 0.071 | 0.598 ± 0.022 | 0.522 ± 0.022 | 0.716 |
| A4tl | 0.567 ± 0.018 | 0.530 ± 0.013 | 0.542 ± 0.016 | 0.313 | 0.542 ± 0.046 | 0.514 ± 0.039 | 0.542 ± 0.016 | 0.100 |
| A1_2_3ll | 0.473 ± 0.021 | 0.341 ± 0.036 | 0.433 ± 0.029 | 0.194 | 0.514 ± 0.039 | 0.456 ± 0.060 | 0.433 ± 0.029 | 0.434 |
| A4ll | 0.416 ± 0.034 | 0.374 ± 0.041 | 0.456 ± 0.020 | 0.168 | 0.496 ± 0.029 | 0.347 ± 0.065 | 0.456 ± 0.020 | 0.082 |
| A1_2_3ulhf | 0.496 ± 0.021 | 0.459 ± 0.026 | 0.376 ± 0.031 | 0.156 | 0.477 ± 0.054 | 0.243 ± 0.059 | 0.376 ± 0.031 | 0.166 |

***** The global properties were calculated with one-way ANOVA test. If the results one-way ANOVA were significance, post-hoc analysis with least significant difference was subsequently applied.

**Table S11. Nodal local efficiency compared between the patients and healthy groups**

| Node name | Glioma grew in the left prefrontal lobe | | | | Glioma grew in the right prefrontal lobe | | | |
|---|---|---|---|---|---|---|---|---|
| | GRE group | non-GRE group | Health group | One-way ANOVA (p value) | GRE group | non-GRE group | Health group | One-way ANOVA (p value) |
| A6m | 0.434 ± 0.090 | 0.589 ± 0.064 | 0.476 ± 0.067 | 0.328 | 0.746 ± 0.040 | 0.658 ± 0.076 | 0.476 ± 0.067 | 0.281 |
| A4hf | 0.624 ± 0.054 | 0.582 ± 0.066 | 0.570 ± 0.062 | 0.835 | 0.634 ± 0.071 | 0.609 ± 0.080 | 0.570 ± 0.062 | 0.947 |
| A4ul | 0.744 ± 0.061 | 0.717 ± 0.064 | 0.705 ± 0.055 | 0.882 | 0.680 ± 0.080 | 0.748 ± 0.045 | 0.705 ± 0.055 | 0.761 |
| A4t | 0.697 ± 0.058 | 0.740 ± 0.050 | 0.711 ± 0.045 | 0.828 | 0.601 ± 0.098 | 0.639 ± 0.104 | 0.711 ± 0.045 | 0.360 |
| A4tl | 0.361 ± 0.080 | 0.400 ± 0.076 | 0.452 ± 0.069 | 0.704 | 0.507 ± 0.086 | 0.638 ± 0.071 | 0.452 ± 0.069 | 0.588 |
| A1_2_3ll | 0.529 ± 0.066 | 0.516 ± 0.078 | 0.430 ± 0.071 | 0.592 | 0.660 ± 0.083 | 0.568 ± 0.099 | 0.430 ± 0.071 | 0.479 |
| A4ll | 0.778 ± 0.037 | 0.707 ± 0.045 | 0.565 ± 0.059 | 0.082 | 0.832 ± 0.047 | 0.767 ± 0.028 | 0.565 ± 0.059 | 0.136 |
| A1_2_3ulhf | 0.700 ± 0.064 | 0.697 ± 0.054 | 0.559 ± 0.056 | 0.132 | 0.667 ± 0.099 | 0.801 ± 0.022 | 0.559 ± 0.056 | 0.465 |
| A1_2_3tonIa | 0.480 ± 0.100 | 0.591 ± 0.078 | 0.576 ± 0.066 | 0.643 | 0.690 ± 0.072 | 0.665 ± 0.088 | 0.576 ± 0.066 | 0.616 |
| A2 | 0.588 ± 0.086 | 0.559 ± 0.076 | 0.564 ± 0.066 | 0.963 | 0.693 ± 0.097 | 0.615 ± 0.099 | 0.564 ± 0.066 | 0.775 |
| A1_2_3tru | 0.366 ± 0.100 | 0.440 ± 0.080 | 0.397 ± 0.068 | 0.816 | 0.725 ± 0.067 | 0.402 ± 0.114 | 0.397 ± 0.068 | 0.083 |
| A6m | 0.376 ± 0.080 | 0.320 ± 0.083 | 0.433 ± 0.070 | 0.567 | 0.662 ± 0.084 | 0.324 ± 0.112 | 0.433 ± 0.070 | 0.111 |
| A4hf | 0.233 ± 0.094 | 0.154 ± 0.059 | 0.325 ± 0.068 | 0.232 | 0.552 ± 0.117 | 0.444 ± 0.110 | 0.325 ± 0.068 | 0.274 |
| A4ul | 0.353 ± 0.088 | 0.192 ± 0.067 | 0.387 ± 0.076 | 0.178 | 0.346 ± 0.111 | 0.315 ± 0.111 | 0.387 ± 0.076 | 0.933 |
| A4t | 0.662 ± 0.039 | 0.780 ± 0.027 | 0.671 ± 0.041 | 0.064 | 0.694 ± 0.047 | 0.797 ± 0.032 | 0.671 ± 0.041 | 0.305 |
| A4tl | 0.677 ± 0.039 | 0.779 ± 0.027 | 0.674 ± 0.042 | 0.111 | 0.691 ± 0.080 | 0.758 ± 0.018 | 0.674 ± 0.042 | 0.382 |
| A1_2_3ll | 0.616 ± 0.079 | 0.531 ± 0.091 | 0.535 ± 0.064 | 0.729 | 0.741 ± 0.065 | 0.602 ± 0.064 | 0.535 ± 0.064 | 0.409 |
| A4ll | 0.611 ± 0.085 | 0.499 ± 0.081 | 0.606 ± 0.061 | 0.523 | 0.491 ± 0.101 | 0.570 ± 0.122 | 0.606 ± 0.061 | 0.338 |
| A1_2_3ulhf | 0.667 ± 0.035 | 0.639 ± 0.056 | 0.498 ± 0.071 | 0.123 | 0.650 ± 0.059 | 0.717 ± 0.053 | 0.498 ± 0.071 | 0.809 |

***** The global properties were calculated with one-way ANOVA test. If the results one-way ANOVA were significance, post-hoc analysis with least significant difference was subsequently applied.
